# Testosterone Administration Moderates Effect of Social Environment on Trust in Women Depending on Second-to-Fourth Digit Ratio

**DOI:** 10.1038/srep27655

**Published:** 2016-06-10

**Authors:** Vincent Buskens, Werner Raub, Nynke van Miltenburg, Estrella R. Montoya, Jack van Honk

**Affiliations:** 1 Department of Sociology/ICS, Utrecht University, Padualaan 14, 3584 CH Utrecht, The Netherlands; 2 Nuffield College, University of Oxford, New Road, Oxford OX1 1NF, United Kingdom; 3 Department of Experimental Psychology, Utrecht University, Heidelberglaan 1, 3584 CS Utrecht, The Netherlands; 4 Department of Psychiatry and Institute of Infectious Disease and Molecular Medicine (IDM), University of Cape Town, 7925 Cape Town, South Africa

## Abstract

Animal research has established that effects of hormones on social behaviour depend on characteristics of both individual and environment. Insight from research on humans into this interdependence is limited, though. Specifically, hardly any prior testosterone experiments in humans scrutinized the interdependency of testosterone with the social environment. Nonetheless, recent testosterone administration studies in humans repeatedly show that a proxy for individuals’ prenatal testosterone-to-estradiol ratio, second-to-fourth digit-ratio (2D:4D ratio), influences effects of testosterone administration on human social behaviour. Here, we systematically vary the characteristics of the social environment and show that, depending on prenatal sex hormone priming, testosterone administration in women moderates the effect of the social environment on trust. We use the economic trust game and compare one-shot games modelling trust problems in relations between strangers with repeated games modelling trust problems in ongoing relations between partners. As expected, subjects are more trustful in repeated than in one-shot games. In subjects prenatally relatively highly primed by testosterone, however, this effect disappears after testosterone administration. We argue that impairments in cognitive empathy may reduce the repeated game effect on trust after testosterone administration in subjects with relatively high prenatal testosterone exposure and propose a neurobiological explanation for this effect.

A growing body of evidence from social neuroscience and neuroeconomics recognizes that testosterone has powerful effects on social behaviour in humans^1^,^2^,^3^. The steroid hormone testosterone is critical in neurodevelopment and has important activational effects on brain and behaviour^2^. Moreover, neurodevelopmental and activational effects interact, as prenatal testosterone relative to estradiol exposure indexed by 2D:4D ratio^4^,^5^ moderates effects of testosterone administration on human social behaviour^6^,^7^,^8^. For instance, a core effect of testosterone is the impairment of cognitive empathy (Theory of Mind, mind reading, cognitive perspective taking)^2^,^6^,^9^,^10^,^11^. Recently, it has been shown repeatedly that relatively high prenatal testosterone exposure primes the deleterious effects of testosterone administration on cognitive empathy^6^,^12^, though the effect has not always been replicated^13^.

Testosterone has also been related to aggressive behaviour in rodents. Research has shown that testosterone underlies many features of human social behaviour but does not trigger aggressive and antisocial behaviour per se. Two approaches on testosterone effects in humans have emerged. An approached not pursued in this article maintains that the hormone induces behaviours that are motivated by seeking dominance and status in social interactions^1^,^14^. Thus, when aggression is a means of generating or securing dominance and status, testosterone should indeed be related to aggression, while in social environments in which dominance and status derive from other and non-aggressive behaviours, the relation between testosterone levels and aggression should disappear. For example, testosterone has been shown to be positively related to fairness in human bargaining behaviour^1^ and to vigilance against the threat of betrayal^15^,^16^. The empirical evidence, though, is still mixed for more complex social and economic interactions between humans^8^,^17^,^18^,^19^. A complementary approach, the one we pursue, focuses on cognitive effects. Testosterone seems to negatively affect cognitive empathy, i.e., the ability to anticipate on others’ behaviour by, for example, reading the mind of others from subtle cues such as recognizing emotions, intentions and feelings from facial expressions^6^,^9^. Importantly, there is also evidence that testosterone not only affects behaviour but that certain behaviours and outcomes of social interactions, in turn, cause higher testosterone levels^1^,^20^. Causality is thus problematic in observational studies on the correlation between testosterone and behaviours. Therefore, experimental designs using placebo-controlled testosterone administration are needed to support causal claims concerning testosterone effects on behaviour in social interactions.

While consensus is emerging that behavioural effects of testosterone in humans and effects of social environment are interrelated^2^, hardly any evidence is available from experimental designs that include the same decisions in different environments. An exception is a recent study showing that the effect of testosterone administration on risk-taking differs between victory and defeat related environments^21^. Our experiment includes fine-tuned variation of the environment tapping into individuals’ cognitive empathic abilities that research has shown to be impaired by testosterone administration^6^,^9^. We use the economic trust game as an experimental paradigm^15^,^19^,^22^,^23^,^24^,^25^. The trust game not only models important features of social and economic interactions, manipulating features of the game also allows to systematically vary the relative demands on cognitive empathy. The feature of the social environment that is varied is whether pairs of subjects play the trust game only once (“one-shot game”) or repeatedly with each other (“repeated game”). The one-shot game models trust problems in relations between strangers, while the repeated game is a model of trust problems in ongoing relations between partners^26^,^27^,^28^. Earlier experimental research on testosterone effects in trust games focuses exclusively on the one-shot game and does not address effects on cognitive empathy^15^,^19^, although the importance of cognitive empathy in the trust game had been recognized already in the first imaging study of this experimental paradigm^25^ (in Supplementary Information, Section 6, we discuss how our results relate to findings from earlier experiments on testosterone effects in one-shot games).

The trust game is played by two subjects, one in the role of an investor, the other in the role of a trustee (Fig. 1). The investor starts and can invest by transferring money to the trustee. The money transferred is increased by the experimenter. Subsequently, the trustee decides on whether and how to share money by a back transfer to the investor. The game models the decision of the investor on whether or not to trust the trustee by investing money and the decision of the trustee to honour or abuse trust by sharing or keeping the money received through the transfer. Both investor and trustee are better off when money is invested by the investor and subsequently shared by the trustee compared to when the investor decides not to invest. Likewise, however, the trustee has an incentive not to share and in that case the investor is worse off after investing than in the case of not investing at all. Due to these features, the trust game is an experimental paradigm for studying both trustfulness, indicated by investments, and trustworthiness, indicated by sharing through back transfers. Trust is often needed, for example, in economic exchange but also in support relations between colleagues in organizations and in social exchange in friendships and intimate social relations^26^,^28^.

If the trust game is played only once between the investor and the trustee, a selfish trustee will abuse trust by not sharing whatever has been received due to an investment of the investor, since sharing would be costly. Anticipating such behaviour of the trustee, the investor has an incentive not to invest in the first place. In the repeated game, incentives and requirements for cognitive empathy are different^27^. In our experiment, the same investor and trustee play six times with each other in the repeated game. Assume that the investor takes into account that the trustee might not be selfish after all and would be willing to share. Such a non-selfish trustee would share in each round of the repeated game. A selfish trustee would certainly not share in the final round, for the same reason that a selfish trustee would not share in the one-shot game. However, even a selfish trustee now has an incentive for reputation building that can affect his behaviour in earlier rounds of the repeated game. Namely, by not sharing already in early rounds, the trustee would reveal selfishness and may induce the investor not to invest anymore in future rounds. Conversely, by sharing in early rounds and not sharing only later on, the trustee could increase own monetary payoffs compared to the situation when the investor does not invest already in early rounds. The investor can likewise increase own monetary payoffs by investing in early rounds as long as the trustee shares, refraining from further investments only after the trustee did not share and towards the end of the repeated game. Thus, in the repeated game, investments of the investor and sharing of the trustee in early rounds can be profitable for both investor and trustee, while investments and sharing break down towards the end of the game (see Supplementary Information, Section 4 for further details). Through conditional behaviour of the investor – investing in early rounds of the game as long as the trustee shares – and reputation building of the trustee, behaviour in the repeated game can substantially differ from behaviour in the one-shot game.

The difference between the one-shot and the repeated game models a key feature of trust problems in human interactions. Repeated interactions in ongoing relations are a feature of many economic and social exchanges. Theory and empirical research, including many experimental studies, also show they are a major driving force of trustfulness and trustworthiness^28^. Trust in the repeated game based on conditional behaviour of the investor and reputation building of the trustee presupposes that both subjects anticipate on each other’s behaviour and requires cognitive empathy more so than in the one shot-game. Anticipating trustee behaviour in the one-shot trust game presupposes (some) cognitive empathy of the investor, while cognitive empathy is not an issue for the trustee in the one-shot game, since the trustee’s payoff after an investment by the investor depends exclusively on the trustee’s own behaviour. In the repeated game, own behaviour in a current round can have repercussions for other’s behaviour in multiple future rounds. However, cognitive empathy is a considerably less demanding task for the trustee than for the investor in the repeated game. Other than for the investor, no cognitive empathy is needed for the trustee in the final round of the repeated game. Moreover, in earlier rounds of the repeated game, the trustee only needs to anticipate on how own behaviour in the current round may affect the behaviour of the investor in future rounds, more precisely, that not sharing in the current round will make the investor very reluctant to invest in future rounds. Cognitive empathy of the investor, however, requires that the investor not only anticipates on how own behaviour in the current round may affect the behaviour of the trustee in the current and future rounds but also anticipates on whether and how the trustee, in turn, might anticipate on the effects of trustee behaviour for subsequent investor behaviour. Cognitive empathy of the investor in the repeated game thus requires an additional step of strategic reasoning. Given that testosterone negatively affects cognitive empathy^6^,^9^, testosterone should reduce the likelihood that investors take this additional step. Therefore, our hypothesis is that testosterone and more particularly the interplay of testosterone administration with high prenatal testosterone-to-estradiol ratio as indexed by 2D:4D ratio^6^ will reduce the difference in investors’ behaviour between the one-shot and the repeated game. Since the trustee’s behaviour is less dependent on cognitive empathy in the one-shot as well as the repeated game, we do not expect such an effect for the trustee, but still report on trustee behaviour below (see also Supplementary Information, Section 3). Note, too, that our hypothesis is exclusively on testosterone effects moderating the difference in trust between one-shot and repeated games rather than testosterone effects on baseline levels of investments in one-shot or repeated games. For such hypotheses, we would also need data about investors’ and trustees’ beliefs on behaviour of others (see Supplementary Information, Section 5).

## Materials and Methods

**Figure 2:**
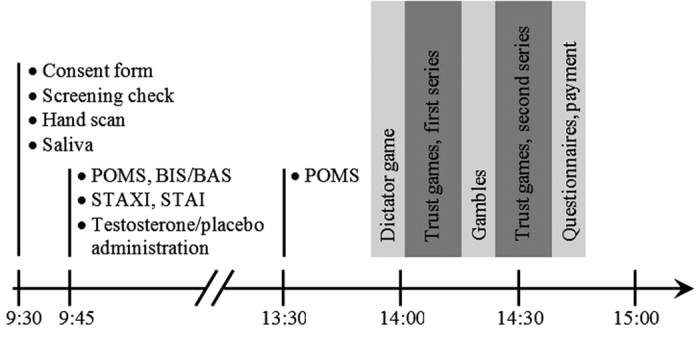
Sequence of events in the experiment. All sessions started at 9:30 in the morning (consent form, final screening, high-resolution scan of the subjects’ right hand, and subjects providing saliva). After filling in POMS-Profile of Mood States, BIS/BAS-behavioural inhibition and behavioural activation questionnaire, STAXI-State-Trait Anger Expression Inventory and STAI-State-Trait Anxiety Inventory, subjects took the substance. At 13:30, they returned to the laboratory, again filling in POMS. They then performed the economic game tasks (dictator game, two series of six trust games, gambles). Some further questionnaires and payments for subjects concluded the experiment.

Our experiment was a double-blind, placebo-controlled study (Fig. 2), employing a between subjects design with a total of 82 young, healthy female subjects (mean age (±s.d.) 22.4 ± 2.2 yr). All subjects provided written informed consent to the protocol of the study that was approved by the medical ethics committee of the University Medical Centre Utrecht. The protocol followed established procedures for experiments on testosterone effects^8^,^14^ and trust games^22^,^23^,^24^. Testosterone and placebo were administered sublingually. Since the parameters (quantity and time course) for inducing neurophysiological effects after a sublingual testosterone administration are known in women but not in men, we exclusively recruited women^29^. Subjects received a single dose of testosterone or placebo four hours before performing the main game tasks. Administration followed the established Tuiten method^6^,^29^. Drug samples consisted of 0.5 mg of testosterone, 5 mg of cyclodextrin (carrier), 5 mg of ethanol, and 5 mL of water. Testosterone was omitted from the placebo samples. Previous experimental research established a 10-fold increase in total plasma testosterone levels 15 min after intake, with testosterone levels returning to baseline within 1.5 h. It is also known from many studies^6^,^29^ that the behavioural effects peak four hours after administration. We checked whether subjects noticed the substance they had received. 73% of the subjects thought they had received placebo. Guesses were unrelated to the actually received substance (Fisher’s exact test, *P* = 0.375, *n* = 81). Since effects of testosterone administration on cognitive empathy seem to depend on priming through prenatal testosterone, we included effects of 2D:4D ratio in the analyses. 2D:4D ratio has been established as an individual marker for differences in prenatal testosterone-to-estradiol ratio^6^. 2D:4D ratio was measured from a scan of the right hand of the subjects, a valid method to measure finger lengths^30^.

Studying how trust in humans is affected by the interplay of biological factors and the social environment required a complex experimental design. The comparison between one-shot and repeated games was crucial. Each subject played six one-shot trust games with six different partners and a repeated game of six rounds with one and the same partner. The two series of games resembled each other as much as possible. The series had to be long enough to ensure sufficient opportunities for conditional behaviour and reputation building in the repeated game. Subjects were informed about behaviour of their partner after each game in both series of games. Half of the subjects started with the one-shot games, the other half with the repeated game. Each subject played in the same role throughout all games. As a result, we had twelve observations per subject, six in the one-shot games and six in the repeated game, with a total of 480 observations from 40 investors and 492 observations from 41 trustees (see Supplementary Information, Section 1 for further details on materials and methods).

Nesting of observations in combination with the necessity to include subject-level controls required hierarchical linear models with random effects at the subject level and fixed effects for whether subjects received testosterone or placebo, whether decisions were made in the one-shot or repeated game, and 2D:4D ratio. Closely related to repeated measure ANOVA models and equivalent to such models in simpler situations, the hierarchical linear models provide straightforward generalizations for our purposes^31^. We report the average marginal effects (AME) of deciding in the repeated game rather than in the one-shot over subgroups of subjects, based on the hierarchical linear models. The effects reflect how much more an investor in a given subgroup invested, on average, when comparing the repeated game with the one-shot game (see Supplementary Information, Section 2 for details).

Altruism may affect behaviour in trust games^23^,^28^ and might depend on testosterone. To measure altruism, subjects played a dictator game^32^ before playing the trust games: each subject received 50 MU and had to choose how much to give to one anonymous other subject in the laboratory. Subjects gave on average 11.4 MU. Giving was neither related to testosterone administration (Mann-Whitney U-test, *P* = 0.424, *n* = 81) nor to 2D:4D ratio (Pearson *r*^2^, *P* = 0.553, *n* = 81). The multivariate model including the interaction between the administered substance and 2D:4D ratio did not explain behaviour in the dictator game (*F*(3, 77) = 0.52, *P* = 0.671). As a further task, and between the two series of trust games, subjects had to make decisions in a series of seven incentivized gambles^33^. This allowed for measuring risk preferences that might be affected by testosterone administration and could be related to behaviour in the trust games. Risk preferences neither varied with the substance administered (Mann-Whitney U-test, *P* = 0.902, *n* = 81) nor with 2D:4D ratio (Pearson *r*^2^, *P* = 0.884, *n* = 81). The model including the interaction between these two variables was likewise insignificant (*F*(3, 77) = 0.27, *P* = 0.850).

## Results

Investments by investors were higher in the repeated than in the one-shot game (AME over all subjects = 1.48, *P* < 0.001). This is consistent with many earlier findings on trust games^27^. On average, subjects invested 6.37 MU in the one-shot and 7.85 MU in the repeated games. Concerning testosterone effects and the interplay of testosterone and the environment, we first mention that we did not replicate the effect found in an earlier study^15^ that investors in the testosterone group invest less in one-shot trust games than investors in the placebo group (see Supplementary Information, Section 6 for details). Note, however, that such effects are anyway not the focus of our present study. According to our hypothesis, testosterone, and more precisely the interplay of testosterone administration and 2D:4D ratio, should decrease the average marginal effect on investments of being in the repeated rather than in the one-shot game. We did not find a difference (Wald test, *P* = 0.605) between the testosterone (AME = 1.30) and the placebo group (AME = 1.65) as such. Comparing the group with above-median 2D:4D ratio (AME = 1.13) with below median 2D:4D ratio (AME = 1.92), we found that the 2D:4D ratio as such had likewise no effect on investments (Wald test, *P* = 0.171). To study the dependence of the effect of testosterone administration on 2D:4D ratio, we included interaction effects between the continuous variable for the 2D:4D ratio, the testosterone condition and whether the game was one-shot or repeated in the multilevel regression analysis (see Model 2 of Table S3 in Supplementary Information, Section 2 for the complete regression model). The significant interaction effects imply that there is a positive effect on investments from being in the repeated game compared to the one-shot game in the placebo condition. The size of this effect does not depend on the 2D:4D ratio. There is also a positive effect of being in the repeated game on investment in the testosterone condition. However, in the testosterone condition this effect becomes smaller with decreasing 2D:4D ratio (indicating increasing relative prenatal testosterone exposure) and disappears at low levels of the 2D:4D ratio. Thus, our moderation hypothesis is confirmed that the positive effect of being in the repeated game rather than in the one-shot game will be reduced by testosterone administration for subjects with low 2D:4D ratio.

**Figure 3:**
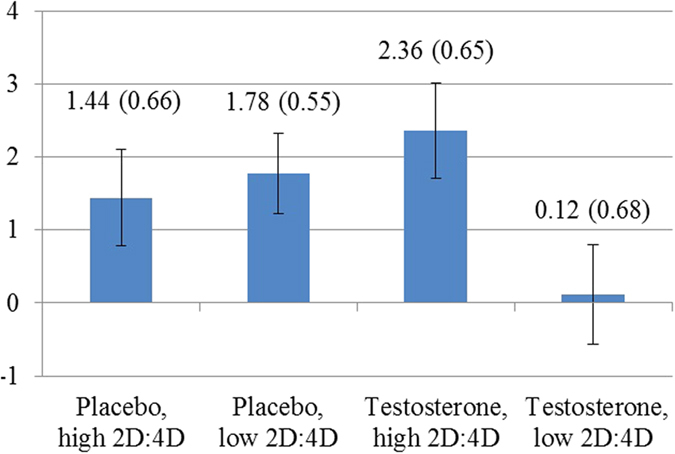
Average marginal effects on investments by the investor of being in the repeated game versus in the one-shot games depending on testosterone administration and 2D:4D ratios. Standard errors of the effect estimated using a hierarchical linear model are indicated in brackets and using error bars. Subjects in the testosterone group with low 2D:4D ratio, indicating relatively high prenatal testosterone exposure, did not invest more in the repeated game than in the one-shot game (*P* = 0.856). Subjects in the three other groups did (from left to right *P* = 0.029, *P* = 0.001, and *P* < 0.001). The effects for all these three groups are also significantly different from the effect for the testosterone group with low 2D:4D ratio.

To facilitate interpretation of the results and qualify the interaction effects with 2D:4D ratio further, we split both the testosterone and placebo group in two subgroups of subjects with either above or below median 2D:4D ratio and compared the average marginal effects of playing the repeated game separately for these four groups (Fig. 3). It then turned out that three out of the four groups had indeed a significant and positive AME (placebo, high 2D:4D ratio: AME = 1.44, *P* = 0.029; placebo, low 2D:4D ratio: AME = 1.78, *P* = 0.001; testosterone, high 2D:4D ratio: AME = 2.36, *P* < 0.001), while this effect did not appear in the group that received testosterone and had low 2D:4D ratio (AME = 0.12, *P* = 0.856). This likewise confirmed our hypothesis and was in line with recent results showing that the effect of testosterone administration on cognitive empathy depends on 2D:4D ratio^6^. When we included controls for whether decisions were made first in the one-shot games and second in the repeated game or the other way round, this had no effect on the results. We also included controls in which of the six games in a series a decision was made as well as interactions with whether such a decision was made in the repeated game or in a one-shot game. Adding these controls did not substantially change the difference between investments in one-shot and repeated games. Due to the feedback between games, subjects adapt their behaviour. We thus checked and established the robustness of our results to controlling for these adaptations. We also checked and established robustness to controlling for various individual characteristics of subjects, in particular, altruism, risk preferences, subjects’ beliefs concerning testosterone administration and effects, salivary testosterone, moods (POMS), behavioural inhabitation and activation (BIS/BAS), anger and anxiety treats (STAXI, STAI), trust, and trustworthiness (see Supplementary Information, Section 1). As an additional test for whether the difference between one-shot games and repeated games was indeed smaller for the group with testosterone administration and low 2D:4D ratio, we analysed the difference between this specific group versus all other subjects together. This difference was indeed significant (Wald test, *P* = 0.020).

**Figure 4:**
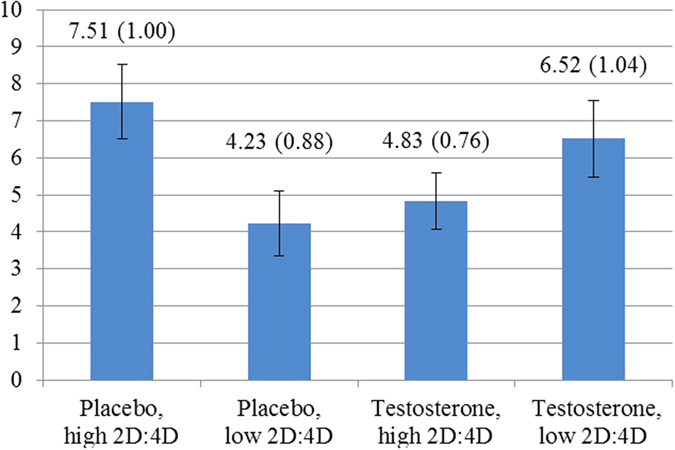
Average marginal effects on sharing by the trustee of being in the repeated game versus in the one-shot games depending on testosterone administration and 2D:4D ratio. Standard errors of the effect estimated using a hierarchical linear model are indicated in brackets and using error bars. Subjects in the placebo group with high 2D:4D ratio, indicating relatively low prenatal testosterone exposure, exhibit a significantly larger repeated game effect than the placebo, low 2D:4D-group and the testosterone, high 2D:4D-group (*P* = 0.004 and *P* = 0.025, respectively).

For the analysis of sharing behaviour of the trustee, we needed to also control for investors’ behaviour because trustees would, on average, share more when they received more. In addition, the relation between investments and sharing might depend on whether the one-shot or repeated game was played. Indeed, if the investor invested nothing, the trustee returned virtually nothing in the one-shot game (0.2 MU on average) and in the repeated game (0.9 MU), while the trustee returned 11.5 MU in the one-shot and 20.5 MU in the repeated game when the investor invested 12 MU. Thus, we did find that trustees shared more in the repeated game. Averaged over all investments by investors, trustees shared 7.3 MU more in the repeated than in the one-shot games. We did not find that administering testosterone had an effect on the difference in sharing between one-shot games and repeated games. The AME for the testosterone group was 5.37 MU and for the placebo group 5.60 MU. The difference between these effects was not significant (Wald test, *P* = 0.804). Also, the 2D:4D ratio did not change the effect of being in the repeated game. Subjects with a low 2D:4D ratio shared 5.21 MU more on average in the repeated game and the subjects with a high 2D:4D ratio shared 5.69 MU more. These groups are again based on a median split of the 2D:4D ratio. The difference was again not significant (Wald test, *P* = 0.537). We then considered the combination of testosterone administration and the 2D:4D ratio (Fig. 4). Here we find, consistent with earlier studies, a significant effect on sharing of being in the repeated game, suggesting that cognitive empathy is less problematic for trustees than for investors. While some of the differences between the groups are indeed significant, we cannot account for these differences in terms of cognitive empathy effects. The highest amount of extra sharing occurred in the group that received placebo and had a high 2D:4D ratio (AME = 7.55). Extra sharing was lower in the groups that either had a low 2D:4D ratio (AME = 4.23) or received testosterone (AME = 4.84), but not both. The extra sharing was again slightly higher in the group with a low 2D:4D ratio that received testosterone (AME = 6.54), which was not significantly different from the other three groups. The difference for the placebo and high 2D:4D group was significantly different from the two middle groups (*P* = 0.004 and *P* = 0.025, respectively).

## Discussion

Including both placebo-controlled substance administration as well as systematic variation of the social environment in an experimental design allowed to establish the moderating effect of testosterone on the difference in trust between one-shot and repeated games. We show that, in healthy young women with relatively high prenatal testosterone exposure (i.e., with low 2D:4D ratio), administration of testosterone moderates the effect of the social environment on trust behaviour. Thus, the interplay of biological factors and characteristics of the social environment affects human interactions, including interactions with important implications for economic and social life. While claims are not new that it is the interplay of biological and social factors, rather than only their separate effects, that shapes behaviour, supporting such claims with experimental evidence requires biological as well as social factors to be varied systematically. Our results correspond to prior research also showing that relatively high prenatal testosterone exposure boosts the negative effect of testosterone administration on cognitive empathy^6^. At present, typical differences in trust between partners and strangers disappear after testosterone administration in subjects with relatively high prenatal testosterone exposure. Defensibly, this is caused by the impairment in cognitive empathy after testosterone administration in these subjects^6^, making them less capable for taking other people’s perspectives.

Our results are well in line with previous studies^6^,^12^. Still, findings should be interpreted with caution. Note that in our sample of 40 investors in the experiment we have only 10 subjects per cell, since we need to interact testosterone administration and 2D:4D ratio. This power issue should not be neglected and makes the theoretical explanation of our results on investor behaviour in terms of our hypothesis even more important. In addition, we propose below a hypothetical neurobiological mechanism that could account for how behaviour in our experiment might have been affected by the interaction between prenatal testosterone exposure and current testosterone administration.

As noted, 2D:4D ratio is interactively shaped by testosterone and estradiol in utero^4^,^5^. Furthermore, many effects of testosterone on social behaviour are thought to arise after metabolism to estradiol by the enzyme aromatase, and the gene RORA, which transcriptionally regulates the enzyme aromatase, is under potential negative and positive feedback of the very sex hormones. Thus, aromatase differs between individuals depending on sex hormone levels^34^. Theorizing on this basis, we argued in our earlier work that the prenatal sex-steroid balance marked by 2D:4D ratio might also be predictive for the rate of metabolism of testosterone into estradiol^7^,^8^. That is, subjects prenatally more strongly primed by testosterone metabolize less testosterone into estradiol, while subjects more strongly primed by estradiol metabolize more testosterone into estradiol. As a result, in subjects relatively highly primed by testosterone prenatally, effects of testosterone administration are mediated by androgen receptors and those prenatally relatively highly primed by estradiol via oestrogen receptors^7^,^8^. Consequently, effects of testosterone administration in subjects with either low or high 2D:4D ratio^6^,^7^,^8^ may thus involve different pathways^7^,^8^, with the oestrogen pathway inducing more female type social behaviours^7^,^8^ and the androgen pathway presently underlying a male-type social-cognitive impairment^6^,^8^. Indeed, typically observed different trust levels in the one-shot and the repeated trust game disappeared after testosterone administration, but exclusively in subjects relatively highly primed prenatally by testosterone. This suggests impairment of cognitive empathy^6^ in terms of social perspective taking. Our aromatase hypothesis is of course speculative and awaits further testing. However, the hypothesis corresponds to current theoretical accounts arguing that the masculinization of brain and behaviour in humans differs from rodents. In rodents, masculinization remarkably involves aromatase of testosterone to estradiol and subsequent effects on estrogen receptors. In humans, more straightforwardly, the androgen receptors seem to underlie masculinization and, as we propose, also impairments in cognitive empathy^35^,^36^.

Remaining questions are whether genomic or fast acting non-genomic mechanisms are involved in the present effects of testosterone on social behaviour, and what brain structures might be involved^37^. Given the four-hour time course of effects we apply and since these effects occur after testosterone levels are normalized for several hours^29^, genomic effects can be presumed. This might especially be the case with an androgen pathway^38^. However, after conversion to estradiol, non-genomic effects might be more likely. Estradiol is even suggested to rapidly act as a neurotransmitter in the brain^39^. This suggests that non-genomic effects after conversion to estradiol might still be involved in our findings on testosterone administration in subjects prenatally relatively highly primed by estradiol^7^,^8^.

In sum, both genomic and non-genomic mechanisms are hypothetically involved in effects of testosterone administration on a broad range of social and emotional behaviours^2^, and their relative involvement might depend on prenatal sex hormone priming. Currently, studies in rodents and humans are underway to get more definitive insights.

Finally, testosterone seems to act acutely on social behaviour by inducing changes in the functional connectivity of the brain^2^. Testosterone and estradiol are thought to act on social behaviour by binding to steroid responsive neurons that occupy a wide, but selective range of regions and nuclei in the brain and interact with other integrative neural mechanisms that affect motivation and emotion^40^,^41^. Anatomical evidence in rodents suggests that these steroid responsive networks interact with the environment filtering and channelling sensory information leading to a specific behavioural response^42^. The left inferior frontal gyrus (IFG) appears to be crucially involved in cognitive empathy^43^ and, recently, it has been shown using pharmacological functional neuroimaging that administration of testosterone reduces the connectivity of the left IFG with the anterior cingulate cortex (ACC) and the supplementary motor area (SMA) during the performance of a task requiring cognitive empathy^44^. This IFG-ACC-SMA network underlies the integration and selection of sensory information for action preparation during cognitive empathic behaviour^45^,^46^,^47^,^48^,^49^, and might thus be part of one of the steroid responsive networks discussed above^40^,^41^. As a next step it would be interesting to use pharmacological functional neuroimaging to investigate how the neural mechanisms by which testosterone acts on trust vary between social environments using one-shot and repeated games.

## Additional Information

**How to cite this article**: Buskens, V. *et al*. Testosterone Administration Moderates Effect of Social Environment on Trust in Women Depending on Second-to-Fourth Digit Ratio. *Sci. Rep.* **6**, 27655; doi: 10.1038/srep27655 (2016).

## Supplementary Material

Supplementary Information

## Figures and Tables

**Figure 1:**
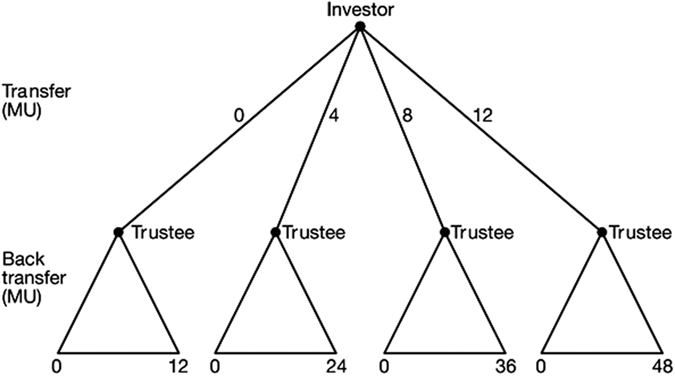
The trust game. Following Kosfeld *et al*.^20^, each subject receives an endowment of 12 monetary units (MU). The investor can invest by transferring 0, 4, 8 or 12 MU to the trustee. The experimenter triples the investment. Subsequently, the trustee is informed about the investment and can send any amount between zero and the total amount available back to the investor. E.g., if the investor has sent 12 MU, the trustee possesses 48 MU (12 plus 36) and can choose any back transfer from 0 to 48 MU. The experimenter does not increase the back transfer. The investor’s final payoff equals the initial endowment minus the transfer to the trustee, plus the back transfer from the trustee. The trustee’s final payoff is the initial endowment plus the tripled transfer of the investor, minus the back transfer to the investor. At the end of the experiment, MU earned are exchanged into real money according to a publicly announced exchange rate (see Supplementary Information, Section 1).
