# Supplementary material for: Testosterone Administration Moderates Effect of Social Environment on Trust in Women Depending on Second-to-Fourth Digit Ratio
Source: Sci Rep. 2016 Jun 10;6:27655. doi: 10.1038/srep27655 (PMC4901316; doi:10.1038/srep27655)
Supplement: Supplementary Information [file srep27655-s1.pdf]

Testosterone Administration Moderates Effect of Social Environment on Trust in Women Depending on Second-to-Fourth Digit Ratio

Supplementary Information

Vincent Buskens, Werner Raub, Nynke van Miltenburg, Estrella R. Montoya, and Jack van Honk

Table of Contents

1. Details on materials and methods
2. Supplementary statistics and results on investors' behaviour
3. Supplementary statistics and results on trustees' behaviour
4. Conditional trust and reputation building in the repeated trust game
5. Additional hypotheses on testosterone effects on trust through cognitive empathy
6. Remarks on earlier research on testosterone effects on behaviour in trust games
7. References

1. Details on materials and methods

Subjects. Subjects were recruited through a database comprising potential subjects and flyers distributed around the Utrecht University campus. Potential subjects were called by the experimenters and were screened for the experiment. All women used standard oestrogen/progestagen oral contraceptives (containing ethinylestradiol and levonorgestrel). We furthermore checked that subjects would be in the 3-week period of using a single-phase contraceptive at the day of the experiment. In this period, menstrual cycle influences are virtually absent[1] and any effects of the contraceptives would be equal for testosterone and placebo condition. Furthermore, we checked that subjects had no (history of) psychiatric disorders or neurological or endocrine abnormalities, did not smoke, and used no medication other than contraceptive agents. One subject was later discovered to have used medication, and was also tested during the stopping week of the contraceptive. This subject was removed from the analyses, since her menstrual cycle was clearly in another phase than for the other subjects. We checked that including this subject would not have changed our findings. A physician was available during the experiment and could be called by the experimenters had that been necessary.

Experimental Procedure. The experiment comprised six sessions, all held at the Experimental Laboratory for Sociology and Economics of Utrecht University. Subjects arrived in groups of 14 (one session had only 12 subjects) at the laboratory at 9:30 in the morning. When entering the laboratory, subjects were assigned a random number between 1 and 14. This number determined whether a subject would be in the testosterone or placebo group and determined the role in the trust games. Subjects with numbers 1 to 7 were investors, subjects with numbers 8 to 14 trustees. Table S1 shows which number received which substance and the design ensures that half of the investors and half of the trustees received testosterone and the other half placebo. Two subjects did not show up in the first session and therefore we missed two subjects, an investor and a trustee, who would have been number 7 and 14 in the session.

Table S1. Administration design based on random subject number received when entering the laboratory.

Subject number	Session 1	Session 2	Session 3	Session 4	Session 5	Session 6
1	Placebo	Testosterone	Placebo	Testosterone	Placebo	Testosterone
2	Placebo	Testosterone	Placebo	Testosterone	Placebo	Testosterone
3	Testosterone	Placebo	Testosterone	Placebo	Testosterone	Placebo
4	Testosterone	Placebo	Testosterone	Placebo	Testosterone	Placebo
5	Placebo	Testosterone	Placebo	Testosterone	Placebo	Testosterone
6	Placebo	Testosterone	Placebo	Testosterone	Placebo	Testosterone
7	--	Placebo	Testosterone	Placebo	Testosterone	Placebo
8	Testosterone	Placebo	Testosterone	Placebo	Testosterone	Placebo
9	Testosterone	Testosterone	Placebo	Testosterone	Placebo	Testosterone
10	Placebo	Testosterone	Placebo	Testosterone	Placebo	Testosterone
11	Placebo	Placebo	Testosterone	Placebo	Testosterone	Placebo
12	Testosterone	Placebo	Testosterone	Placebo	Testosterone	Placebo
13	Testosterone	Testosterone	Placebo	Testosterone	Placebo	Testosterone
14	--	Testosterone	Placebo	Testosterone	Placebo	Testosterone

Subjects first filled in the consent form. Subsequently, use of contraceptives and the use of other medicines, drugs, and alcohol was checked again. Then, a right-hand scan was used for 2D:4D ratio measurement and subjects' saliva was collected for baseline testosterone level measurement. Also, subjects completed questionnaires on their mood[2] (Profile of Mood States – POMS), behavioural inhibition and behavioural activation[3] (BIS/BAS), the State-Trait Anger Expression Inventory[4] (STAXI) and the State-Trait Anxiety Inventory[5] (STAI). Subjects were assigned randomly to the testosterone or placebo condition, using tubes with either testosterone or a placebo. Tubes were differently labelled, without the experimenters knowing which label belonged to which condition. In this way, a double-blind design was ensured. Testosterone and placebo were administered sublingually at the laboratory.

At 13:30, subjects returned to the laboratory. Subjects again filled in the moods questionnaire. Then, subjects read the instructions (all instructions are available from the authors upon request) for the dictator game. Each subject played one dictator game[6] with an anonymous random subject in the respective session. For the dictator game as well as subsequently for the trust games, subjects were truthfully informed about matching procedures and that they would not come to know the identity of the other subjects with whom they were matched during the experiment. Subjects knew that they would play every game with a new partner, but did not know further details of the scheme in Table S1. Sessions 1, 3 and 5 started with the one-shot games, sessions 2, 4 and 6 with the repeated

games. The matching was systematic in the sense that subject number 1 always played the six one-shot games subsequently with subjects 8, 14, 13, 12, 11, and 10 and the repeated game with subject 9. Subject 2 played the one-shot games with 9, 8, 14, 13, 12, and 11 and the repeated game with subject 10; and so on. This design ensures that subjects would interact with about equal probabilities with other subjects who received placebo or testosterone.

After the dictator game was played, subjects received instructions on the first series of trust games (see Figure 1). To avoid priming of subjects towards trust or cooperation types of sentiments, instructions used neutral terminology rather than labels such as "trust". At any time, subjects could ask questions to the experimenter, but this was hardly ever necessary. At this point, subjects also learned whether they would be investor or trustee in the remainder of the experiment. After reading the instructions, subjects answered a series of 12 questions to check understanding of instructions. More than 75% of the subjects answered nine or more questions correctly. This indicated reasonable understanding because subjects, rather than merely answering multiple choice questions, had to calculate precise earnings under different investor and trustee behaviours. After each question, subjects who provided wrong answers obtained feedback about the correct solutions. Less than 15% of the subjects made more than one mistake on the final six questions. We reanalysed the data excluding the 15 subjects who made more than three mistakes over all questions. This did not affect our results.

After everyone finished reading, subjects played the first series of six trust games. Each subject played the six one-shot games with six different partners and played the repeated game with still another partner (with the exception of subjects in the session with 12 subjects who played the repeated game with one of the six partners from the one-shot games but without knowing which of these partners). In each of the trust games, subjects were immediately and truthfully informed about the behaviour of the partner. z-Tree software was used for presenting the trust games and implementing decisions on computer screens[7]. To interrupt the task between the two series of games and to measure risk preferences, subjects completed a task involving lotteries similar to an established method[8]. Thereafter, subjects read a brief instruction explaining the difference between the first and the second series of trust games and then played the second series. Subjects were informed about their earnings from the dictator game and the gambles only after all trust games had been played. MU were exchanged for Euros at a rate of 25 MU to 1 Euro. Subjects earned between 13.35 and 41.10 (26.58 on average) Euros from the games and gambles, showing that considerable incentives

were involved in the experiment. In addition, subjects received 25 Euros for participation. Subjects could donate (part of) their earnings to a charity. Concluding the experiment, subjects answered a series of questions. First, subjects had to answer questions related to a series of moral dilemmas; second, some basic demographics were asked; third, subjects answered a selection of questions on general trust[9]. Finally, subjects answered questions on their beliefs. First, beliefs on whether they had received testosterone or placebo themselves were measured by a forced choice of their best guess whether they had received testosterone or placebo. Second, subjects answered questions on what they thought testosterone would matter for investor behaviour in one-shot games, investor behaviour in repeated games, and also trustee behaviour in one-shot as well as repeated games.

Digit Ratio Measurement. When conducting the scan, we ensured that details of major creases could be seen. Lengths of the second and fourth digits were measured from the ventral proximal crease of the digit to the fingertip, using Adobe Photoshop measurement-precision tool. When there was a band of creases at the base of the digit, measurement was taken from the most proximal crease. All scans were measured twice by the same researcher with one week in between. We obtained an absolute agreement interclass correlation between the two measurements of 0.83 ($P < 0.001$), an acceptable reliability and common in the literature[10].

Salivary Testosterone Measurement. Baseline testosterone was measured in saliva[11]. Saliva samples were collected just before administration of testosterone and placebo. Laboratory analyses were performed by the Department of Biopsychology at the University of Dresden (Dresden, Germany). Salivary testosterone levels varied between 0.6 and 234 pg/ml (mean 11.2; s.d. 26.4, $n = 80$). Unfortunately, one subject did not provide a saliva sample. We log-transformed salivary testosterone levels because of the skewedness of the distribution. Consistent with results from other studies[11], salivary testosterone was unrelated to 2D:4D ratio ($\rho(78) = 0.059$, $P = 0.60$). There was no difference in salivary testosterone between testosterone and placebo group (T-test, $P = 0.84$) and salivary testosterone did not confound the effects in our main analyses.

Questionnaires. The 37-item shortened version of the Profile of Mood States-POMS[2] was used twice (morning and afternoon measurement) to index possible effects of testosterone on

anger, anxiety, fatigue, vigor, depression, and tension. Items were measured on a scale from –100 to 0 up to +100. The means for the subscales were used to construct the values for the different moods. Mann-Whitney U tests on the 81 subjects showed, in the afternoon, a weakly significant difference for fatigue ($P = 0.037$) between testosterone and placebo, while the other mood indicators did not vary between the groups (anger: $P = 0.103$; anxiety: $P = 0.071$; vigour: $P = 0.141$; depression: $P = 0.183$; tension: $P = 0.239$). Scores on several moods significantly decreased between the morning and afternoon session: anger (change by 0.58, $P = 0.006$), anxiety (change by 0.83, $P < 0.001$), depression (change by 0.75, $P < 0.001$), and tension (change by 0.82, $P < 0.001$). These changes did not seem to be related to the administration of testosterone because changes were similar for the placebo and the testosterone group (anger change placebo group: 0.59, testosterone group: 0.57; anxiety change placebo group: 0.96, testosterone group: 0.70; depression change placebo group: 0.72, testosterone group: 0.78; tension change placebo group: 0.64, testosterone group: 1.00). The other indicators did not change significantly (fatigue: $P = 0.051$; vigour: $P = 0.211$). Many earlier studies that used the same methodology have shown that testosterone does not have a direct effect on subjects' moods[11]. To check for possible confounding of our effects due to moods, we included controls for main effects of moods measured in the afternoon as well as interactions with whether subjects played one-shot or repeated games. This did not affect our results.

In the morning, we also measured additional subject traits, namely, behavioural inhibition (BIS), behavioural activation (BAS)[3], anger trait, and anxiety trait[4,5]. Mann-Whitney U tests on the 81 subjects showed that none of these traits differs between testosterone and placebo group (BIS: $P = 0.341$; BAS: $P = 0.557$; anger: $P = 0.712$; anxiety: $P = 0.626$). In addition, we did not detect any main effects or interactions with testosterone administration or playing one-shot or repeated games in our analyses.

At the end of the experiment, we asked questions about the extent to which subjects trusted people in general (generalized trust), people they knew already longer (relation-based trust) and the extent to which subjects assessed themselves as trustworthy (seven-point scales)[9]. To avoid priming subjects towards trust issues, these questions were not asked at the beginning of the experiment. Answers to these questions are thus largely endogenous and can therefore not be considered independent measures of tendencies to trust. Still, we checked whether our main results were robust to controlling for these variables. Subjects with more generalized

trust turned out to invest slightly more in the repeated games than subjects with lower generalized trust. The trust measures did not have effects when added to our main analyses. Moreover, adding them to the analyses did not affect our results.

Altruism and Risk Preferences. While altruism and risk preferences did not vary between testosterone and placebo group, they might still explain differences in behaviour and thus interfere with our results. Therefore, we conducted additional analyses, including decisions in the dictator game and the gambles as main effects as well as interactions with the administered substance and whether subjects played one-shot or repeated games. Altruistic subjects invested slightly more in all games and risk seeking subjects invested slightly more in the one-shot games. However, controlling for these effects did not change anything in the main results.

Belief Effects. Subjects' beliefs about having been administered testosterone and their beliefs about effects of testosterone can affect their behaviour due to the "folk hypothesis" that testosterone might make people more aggressive[12]. At the end of the experiment, after having played both series of trust games, subjects indicated whether they believed to have had received testosterone or placebo. Guesses were not related to what subjects had actually received (Fischer's exact test: $P = 0.375$). When asking subjects why they thought they had received one or the other substance, most subjects stated explicitly that they did not feel anything and those who stated to feel something often received the placebo. We controlled for whether subjects behaved differently if they believed that they received testosterone by adding a main effect for this belief and an interaction with whether they played a one-shot or repeated game. No effects of the belief were found on investor behaviour.

To further strengthen our test whether or not beliefs of subjects might have affected behaviour, we asked them what they believed the effects of testosterone would be on behaviour of investors and trustees in one-shot and repeated games. These questions had four answering categories: whether investors/trustees would invest/share more or less, whether there would be no effect, or whether they did not know. On all four questions, between 35% and 48% of the subjects indicated that they did not know what the effect would be or that they thought there would be no effect. Concerning investments of the investors, about half of the remaining subjects thought testosterone would lead to more and the other half that it would lead to less investments. Concerning sharing, a large majority of the subjects who

thought they had an idea about the effect, thought a trustee who had received testosterone would share less, irrespective of whether the game was a one-shot or a repeated game. Based on these beliefs about behaviour and the belief about the substance subjects thought they had received themselves, we constructed a variable indicating whether a subject, based on the combination of her beliefs, might invest more or less. We added this variable into our analyses. It turned out that the variable had no effect on investments, nor did adding this variable affect our main findings.

Statistical analysis. We used maximum likelihood estimation of hierarchal linear regression models[13] in which investments are treated as interval variables. Investments can only have values 0, 4, 8, and 12. A more complex hierarchical interval regression model for investments taking into account that the variable had only these four possible values yielded virtually the same results. Dummy variables for whether subjects received either testosterone (0) or placebo (1), whether the decision was in a one-shot (0) or a repeated game (1) were included as fixed effects. 2D:4D ratio was included as a continuous variable as well as all the two-way and three-way interactions between these three variables. Subjects' decisions are the units of analysis. We added a random effect for each subject to account for the nesting of observations within subjects. The flexibility of the hierarchical linear models allowed us to add various controls to the main models to investigate the robustness of our results as reported. Full models for the most important analyses and further details can be found in the next section.

2. Supplementary statistics and results on investors' behaviour

Table S2. Investments (investors' transfers) in the trust game for the one-shot games and the repeated games (n = 40).

	2D:4D ratio (indexing relative prenatal testosterone exposure)					
	High		Low		All	
Treatment group	One-shot	Repeated	One-shot	Repeated	One-shot	Repeated
Placebo	6.67	7.75	5.74	7.74	6.10	7.75
Testosterone	6.06	8.67	7.33	7.19	6.67	7.96
All	6.33	8.26	6.39	7.52	6.37	7.85

Each cell in Table S2 represents the mean amount invested over all observations in that cell. In accordance with our main result, the smallest difference between one-shot and repeated games is found for subjects in the testosterone treatment group with low 2D:4D ratio (relatively high prenatal testosterone exposure) (7.33 versus 7.19). This difference is not equal to the average marginal effect of playing the repeated game from the multivariate analyses displayed by the rightmost bar in Figure 3, because not all relevant controls are taken into account in Table S2.

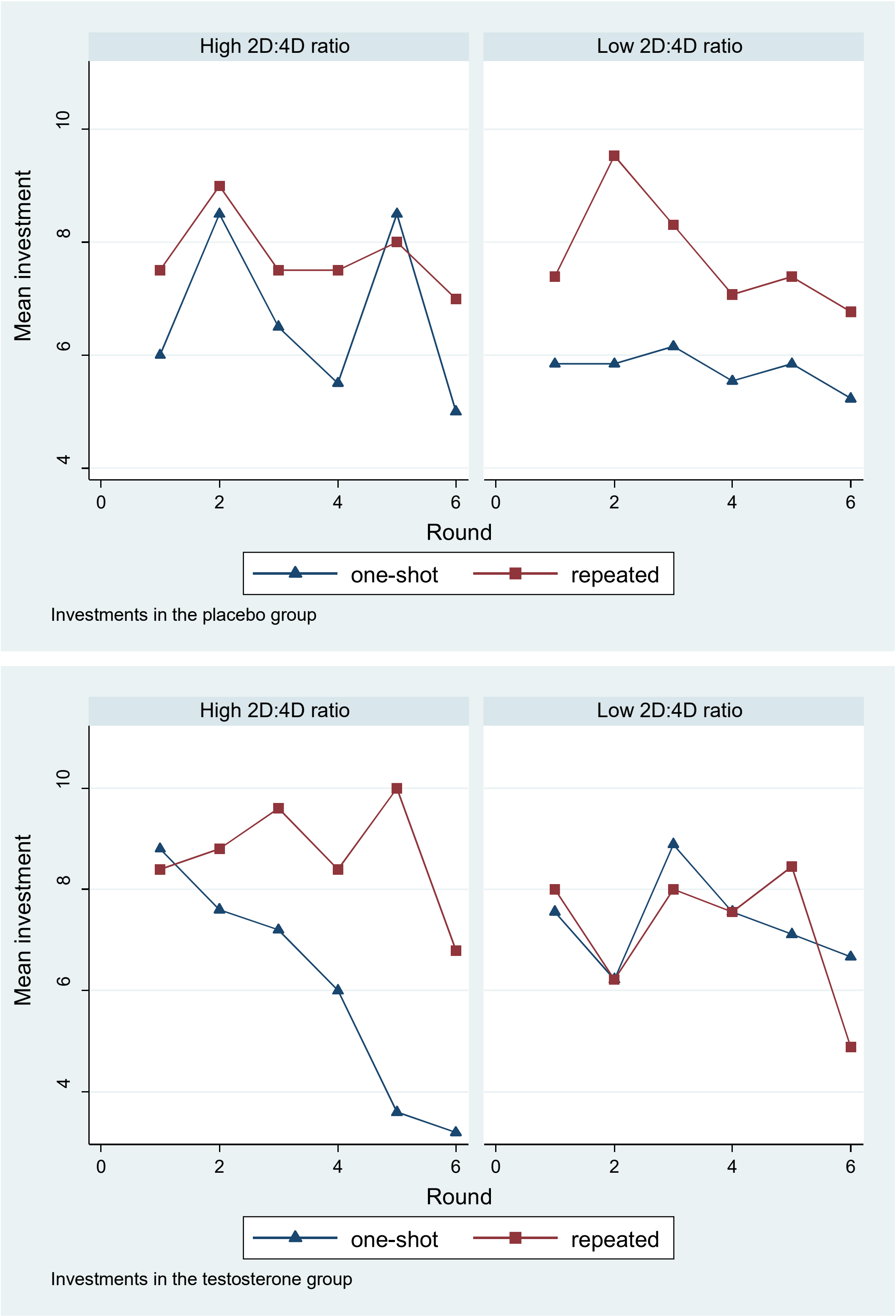

Figure S1. Investments (investors' transfers) in the different conditions over the rounds of the games.

Table S3. Hierarchical linear regression model on investments (investors' transfers) with random effects at the subject level with and without 2D:4D ratio included (480 observations clustered within 40 subjects).

	Model 1 (without 2D:4D)			Model 2 (with 2D:4D)		
	Coeff.	S.E.	*P*	Coeff.	S.E.	*P*
Testosterone	0.57	0.85	0.503	0.28	0.84	0.741
Repeated	1.65	0.47	0.000	1.59	0.48	0.001
2D:4D ratio				37.29	21.98	0.090
Testosterone * Repeated	-0.35	0.68	0.605	-0.26	0.69	0.705
Testosterone * 2D:4D				-74.02	31.56	0.019
Repeated * 2D:4D				-8.05	18.02	0.655
Testosterone * Repeated * 2D:4D				55.29	25.88	0.033
Constant	6.10	0.59	0.000	-29.73	21.12	0.159
Subject level standard deviation	2.22	0.31		2.12	0.30	
Observation level standard deviation	3.73	0.13		3.70	0.12	
Log likelihood		-1346.02			-1341.19	

Adding 2D:4D ratio and its interaction effects entails a significant improvement of the model (Wald test: $P = 0.043$).

Table S4. Overview of robustness analyses: investments (investors' transfers). The first column indicates the model and the additional controls included in the respective extension of Model 2 in Table S3. The first four data columns provide the average marginal effect (AME) of investments in the repeated game rather than in the one-shot game for subjects in the placebo, high 2D:4D (P, HD) group; the placebo, low 2D:4D (P, LD) group; the testosterone, high 2D:4D (T, HD) group; and the testosterone, low 2D:4D (T, LD) group. Note that the average marginal effect of, e.g., the (P, HD) group reports the average of the marginal effects over all subjects who received the placebo and have an above-median 2D:4D ratio based on an analysis in which the 2D:4D ratio is included as a continuous variable. The final column provides the *P*-value, based on a Wald test, establishing the robustness of the moderation effect of testosterone by showing that the (T, LD) group had a smaller AME than the other three groups combined. All robustness checks related to including extra controls involve adding main effects as well as interactions with the dummy variable for whether the decisions were in one-shot or repeated games and the dummy for testosterone administration to ensure that these additional controls indeed did not account for the difference between the one-shot and the repeated games explained through testosterone.

**Model**	**P, HD**	**P, LD**	**T, HD**	**T, LD**	**Wald test**
Model 2 in Table S3	1.44	1.78	2.36	0.12	0.020
Model 2 + controls related to round (continuous), last round, ordering of treatments	1.47	1.77	2.95	-0.77	0.000
Model 2 + controls related to round, last round, ordering of treatments + interactions of testosterone administration with round and last round	1.47	1.77	2.95	-0.77	0.000
Model 2 with a more complex random structure, estimating different random components per subject for the two series of six games[1]	1.44	1.78	2.36	0.12	0.227
Model 2 using a hierarchical interval regression model to account for the non-interval character of the dependent variable[2]	2.25	2.56	3.41	0.30	0.028
Model 2 excluding subjects with less than 9 out of 12 correct answers to control questions	1.87	2.22	2.46	0.13	0.007
*All remaining analyses include Model 2 + main effect for the variable mentioned as well as interactions with Testosterone, Repeated, and Testosterone*Repeated*					
Round, using dummies for every round	1.44	1.78	2.36	0.12	0.015
Extent of sharing of the trustee in the previous round[3]	0.60	1.07	1.33	-0.72	0.015
Altruism (amount given in dictator game)	1.59	1.69	2.26	0.23	0.031
Risk preferences (number of gambles chosen)	1.38	1.82	2.33	0.15	0.020
Belief of a subject that she obtained testosterone	1.44	1.78	2.35	0.13	0.021

Hypothetical belief of a subject what she should do, given what she believed that she had obtained (testosterone or placebo)	1.46	1.82	2.36	0.12	0.018
Salivary testosterone (logarithm)	1.68	1.63	2.33	0.15	0.022
Mood: anger	1.59	1.69	2.28	0.21	0.030
Mood: anxiety	1.47	1.76	2.17	0.33	0.050
Mood: fatigue	1.24	1.90	2.34	0.14	0.021
Mood: vigor	1.33	1.85	2.52	-0.06	0.007
Mood: depression	1.45	1.77	2.21	0.28	0.039
Mood: tension	1.57	1.70	2.18	0.32	0.049
Behavioural inhibition	1.44	1.78	2.29	0.19	0.028
Behavioural activation	1.30	1.87	2.25	0.24	0.031
Trait: anger	1.48	1.75	2.13	0.38	0.058
Trait: anxiety	1.51	1.74	2.10	0.40	0.062
Generalized trust	1.42	1.79	2.25	0.24	0.030
Relation-based trust	1.49	1.75	2.35	0.13	0.020
Own trustworthiness (self-assessment)	1.33	1.85	2.34	0.14	0.018

[1] In this model, we tested whether the random effect for subjects is different for one-shot games compared to repeated games, while we assumed that these two random effects are the same in our main model. The model shows no evidence that the assumption that the two random effects are the same is violated in our data. The two random effects have very similar estimates. In addition, the effect sizes are very similar compared to those from Model 2 in Table S3. As a result, this more complex model does not fit the data better than the simpler model, but only provides much more conservative estimates. Because the more complex model shows that there is no reason to fall back on the more conservative estimates, we stick to the simpler model for further analyses and our main conclusions.

[2] Note that the scale of the dependent variable slightly changes due to moving to interval regression, which increases all effect sizes to a similar extent.

[3] Sharing has to be operationalized here in a way so that it is comparable between different investment levels. Therefore, we use the amount back transferred divided by twice the investment as an indicator for sharing. This proportional sharing equals 1 if the trustee divides the total amount of points equally. Adding sharing as an explanation for investments decreases all the coefficients because sharing turns out to be an important predictor for investments in the subsequent round. The effect of sharing is slightly larger in repeated interactions than in one-shot interactions, but only for subjects who did not receive testosterone.

3. Supplementary statistics and results on trustees' behaviour

This section provides supplementary statistics on trustees' sharing behaviour. We first present aggregate descriptive results and subsequently we provide the regression table of the analysis that led to the average marginal effects of being in the repeated game in Figure 4 (Model 4 in Table S6 below).

Table S5. Sharing (trustees' back transfers) in the trust game for the one-shot games and the repeated games (*n* = 41).

	2D:4D ratio (indexing relative prenatal testosterone exposure)					
	High		Low		All	
Treatment group	One-shot	Repeated	One-shot	Repeated	One-shot	Repeated
Placebo	5.58	16.60	6.50	12.14	6.11	14.02
Testosterone	7.73	13.94	4.05	11.98	6.56	13.32
All	6.99	14.87	5.55	12.07	6.35	13.64

Each cell in Table S5 represents the mean back transfer over all observations in that cell. The average marginal effects of the partner condition cannot be inferred from Table S5, because back transfers are strongly dependent on investments by the investor.

Note that entries in corresponding cells in Tables S2 and S5 do not refer to the same games, because investors in the placebo group played with trustees in the testosterone group as well as in the placebo group and similarly for investors in the testosterone group.

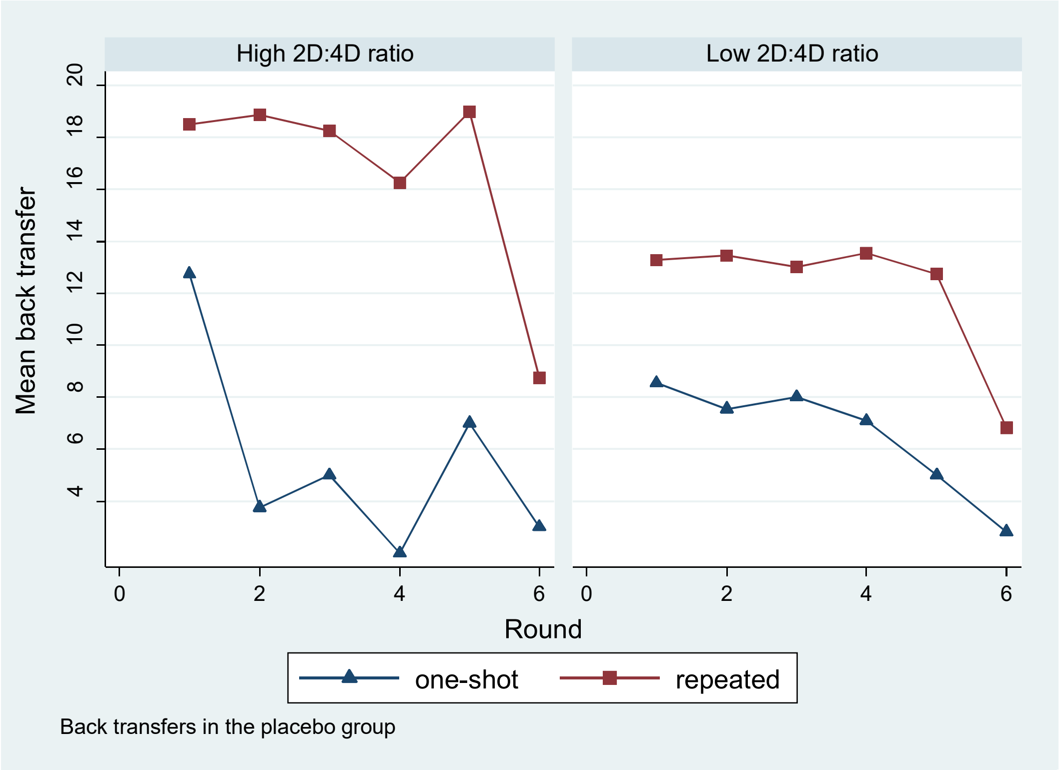

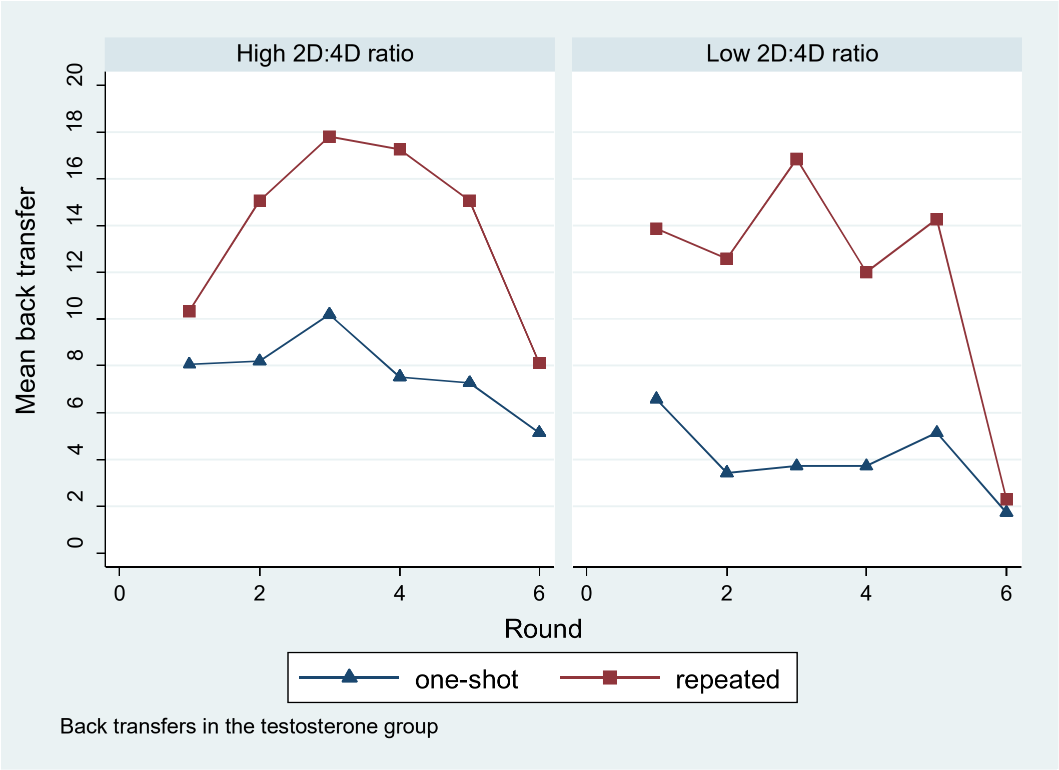

Figure S2. Trustees' back transfers (sharing) in the different conditions over the rounds of the games.

Table S6. Hierarchical linear regression model on trustees' back transfers (sharing) with random effects at the subject level (492 observations clustered within 41 subjects).

	Model 3 (without 2D:4D)			Model 4 (with 2D:4D)		
	Coeff.	S.E.	*P*	Coeff.	S.E.	*P*
Testosterone	0.12	1.07	0.912	-0.39	1.11	0.726
Repeated	3.20	1.34	0.017	3.67	1.36	0.007
2D:4D ratio				-25.83	27.46	0.347
Testosterone * Repeated	-0.22	0.95	0.820	-0.18	1.01	0.861
Testosterone * 2D:4D				75.88	35.62	0.033
Repeated * 2D:4D				52.75	25.34	0.037
Testosterone * Repeated * 2D:4D				-84.99	32.61	0.009
Investment = 0	Ref.			Ref.		
Investment = 4	4.50	1.04	0.000	4.31	1.04	0.000
Investment = 8	8.35	1.05	0.000	8.23	1.05	0.000
Investment = 12	11.91	1.07	0.000	11.77	1.06	0.000
Repeated * Investment = 0	Ref.			Ref.		
Repeated * Investment = 4	-0.89	1.60	0.580	-0.69	1.59	0.666
Repeated * Investment = 8	3.02	1.65	0.068	2.87	1.65	0.082
Repeated * Investment = 12	5.31	1.54	0.001	5.14	1.54	0.001
Constant	-0.35	1.07	0.744	24.58	26.42	0.352
Subject level standard deviation	2.64	0.40		2.56	0.39	
Observation level standard deviation	5.24	0.17		5.20	0.17	
Log likelihood		-1541.82			-1537.08	

Adding the 2D:4D ratio and its interaction effects entails a significant improvement of the model (Wald test: $P = 0.048$).

4. Conditional trust and reputation building in the repeated trust game

To see how conditional behaviour and reputation building can induce trustfulness and trustworthiness in the repeated game, consider a simplified version of the trust game. In the one-shot game, investor and trustee both start with initial endowments P both for the investor and for the trustee. The investor can either transfer or not transfer her initial endowment. If the investor does not invest, the game ends with payoffs P for investor and trustee. If the investor does invest, the investment is multiplied by some factor $m > 1$ and the trustee receives mP. Subsequently, the trustee can choose between sharing by sending $(m + 1)P/2$ back to the investor or keeping $mP + P$ for herself. When the trustee shares, the investor's and the trustee's final payoffs from the one-shot game are $R = (m + 1)P/2 > P$. Otherwise, the trustee's final payoff is $T = mP + P > R$, while the investor's final payoff is $0 < P$.

Assume now that the investor believes that for some reason the trustee might share in the one-shot game. For example, not sharing might create a bad conscience for the trustee that outweighs the trustee's gains $T - R = (m + 1)P/2$ from not sharing. The investor maintains some probability $\pi > 0$ that she interacts with such a "good" trustee and assumes that she interacts with a "bad" trustee who would not share in the one-shot game with the complementary probability $1 - \pi$. The investor cannot directly observe whether the trustee is good or bad but can try to infer this from the trustee's behaviour during the game. Clearly, the bad trustee would not share at least in the final round of the repeated game and hence an investor trying to maximize her payoffs and anticipating on the trustee's behaviour would invest in the last round only if she considers it sufficiently likely that the trustee would share even in the one-shot game.

Also, assume that for some reason the investor has concluded that she interacts with a bad trustee. It is then clear that the investor will never invest in future rounds of the repeated game. This can be seen as follows. The bad trustee would not share in the final round, so there would be reason not to invest in that round. Then, however, there is likewise no reason to invest in the last but one round: since the investor will anyway not invest (and the bad trustee would not share) in the final round, the bad trustee has no reason to share in the last but one round, since sharing will not affect future behaviour of the investor. This reasoning likewise applies for earlier rounds, back to the round in which the investor concludes that she interacts with a bad trustee.

However, the situation is different in earlier rounds of the repeated game when the investor is unsure whether she interacts with a good or a bad trustee. Namely, if the investor invests in some round of the repeated game that is not the final round, the trustee might share for one of two very different reasons. First, the trustee may be a good one who would share also in the one-shot game. Or, second, the trustee may be a bad one who would not share in the one-shot game but follows an incentive for reputation building. The bad trustee knows that if she does not share, the investor can infer for sure that she interacts with a bad trustee and will thus never invest again in future rounds. On the other hand, if the trustee shares, the investor remains uncertain about the trustee and may invest again in the next round. Conversely, the investor can anticipate on such behaviour of the trustee and may therefore be inclined to indeed invest. Thus, the investor tacitly induces the trustee to share through conditional behaviour such that investing in future rounds depends on sharing by the trustee in the current round and the investor tries to learn about the kind of trustee she interacts with from the trustee's behaviour in previous rounds, taking the trustee's incentives for reputation building into account. The trustee, on the other hand, balances the long-term effects of her reputation – if the trustee does not share in the current round, the investor will not invest in future rounds – and the short-term incentives for not sharing, taking into account that the investor anticipates on this balancing. It can be shown that this interplay of investor and trustee behaviour can involve investments and sharing in the early rounds of the repeated game and indeed for many rounds, with no investments or no sharing only in the very final rounds of the repeated game, so that each player maximizes own payoffs, given the behaviour of the other player. A remarkable feature is that even a small initial probability of interacting with a good trustee suffices to induce such a pattern[14]. Thus, even a small initial probability of interacting with a good trustee can suffice to induce a pattern of behaviour in the repeated game – investments and sharing in many rounds – that can differ drastically from behaviour in the one-shot game.

Remark: Our study provides evidence supporting the conjecture that testosterone plays a role in complex human interactions involving conditional behaviour and reputation building. It would be an interesting extension to study testosterone effects in a social environment with reputation deriving not only from repeated interactions between the same two partners but also from third-party information in a network in which an investor receives information about the trustee's behaviour also from other investors who interacted with the trustee. It is a well-known regularity that such network effects exist but are weaker than the effects due to

repeated interactions between the same two partners[15]. Note that cognitive empathy, compared to a repeated game between the same investor and trustee, is still more demanding for interactions in such a network, suggesting that testosterone effects might again play a role.

5. Additional hypotheses on testosterone effects on trust through cognitive empathy

Our hypothesis focuses on how testosterone affects differences in subjects' behaviour between the one-shot and the repeated game. It is conceivable to come up with additional hypotheses on how testosterone affects behaviour in trust games due to testosterone effects on cognitive empathy. The investor's payoff in the one-shot game after an investment depends on subsequent behaviour of the trustee, while the trustee's payoff in the one-shot game after an investment by the investor depends exclusively on the trustee's own behaviour. In the one shot-game, therefore, cognitive empathy is not an issue for the trustee. Hence, one would expect testosterone effects through cognitive empathy on investor behaviour in the one-shot game, but not on trustee behaviour. The argument why no effects of testosterone on trustee behaviour through cognitive empathy are expected for the one-shot game also shows that hypotheses on testosterone effects on cognitive empathy have to be distinguished from conjectures concerning effects of testosterone on rational and selfish behaviour, since conjectures on such effects would imply effects, too, on trustee behaviour in the one-shot game. With respect to the repeated game, the trustee's payoff in the final round, given an investment by the investor, again depends only on the trustee's own behaviour. Thus, one would again expect no testosterone effects through cognitive empathy on trustee behaviour in the final round, while one would expect such effects on trustee behaviour in earlier rounds and also on investor behaviour throughout all rounds of the repeated game. However, predictions on how testosterone affects investor behaviour in the one-shot and in the repeated game and how testosterone affects trustee behaviour in early rounds of the repeated game require additional assumptions about the beliefs of subjects, namely, their beliefs about characteristics of the partner and the partner's future behaviour. Only then would it be possible to derive predictions on whether cognitive empathy affects the likelihood of investments and sharing positively or negatively. Our data do not comprise measurements or proxies for such beliefs. Moreover, it should be observed that testing such predictions would be hampered by power problems due to few observations. Note that our hypothesis on how testosterone affects differences in subjects' behaviour between the one-shot and the repeated game does not require additional assumptions on subjects' beliefs about the partner and that more observations are available for testing this hypothesis.

6. Remarks on earlier research on testosterone effects on behaviour in trust games

We identified two earlier studies on testosterone effects on behaviour in trust games[16,17]. Both studies are on behaviour in one-shot games exclusively. One of these studies is an exploratory study on testosterone effects and oestrogen effects on behaviour in a variety of economic games and a task measuring risk aversion, with the trust game as one of the economic games[17]. Details of the design of the trust game differ somewhat from our study and there are quite some design differences with respect to study population and testosterone administration. Each subject played once in the role of the investor and once in the role of the trustee. There were no significant difference between the 3 treatment groups (testosterone (n = 67), oestrogen (n = 66) and placebo (n = 67)) with respect to investor behaviour or trustee behaviour. The other study[16] is very similar to ours in terms of study population and testosterone administration. Each subject (n = 54) played once in the investor and once in the trustee role. A major design difference is that subjects in the trustee role were always told that the investor had invested the maximum amount. The authors develop hypotheses based on the notion that testosterone induces a concern for status. They argue that investor status is threatened by abuse of trust, i.e., not sharing by the trustee. Hence, it is predicted that investors in the testosterone group would invest less than investors in the placebo group. This prediction is confirmed by the data. Concerning trustee behaviour, it is argued that not sharing is beneficial in the short term but entails long-term costs by damaging a subject's reputation. Thus, via the effect on status concerns, testosterone is predicted to induce more sharing. This prediction is likewise confirmed by the data. The results of the first study are consistent with those in our study, while those of the second study are at odds with ours. We did not identify a significant effect of testosterone administration on investors' or on trustees' behaviour in the one-shot games and investments are even slightly higher in the testosterone condition than in the placebo condition.

7. References

1. Aarts, H. & Van Honk, J. Testosterone and unconscious positive priming increase human motivation separately. *Neuroreport* **20**, 1300–1303 (2009).
2. Shacham, S. A shortened version of the Profile of Mood States. *J. Pers. Assess.* **47**, 305–306 (1983).
3. Carver, C. S. & White, T. L. Behavioral inhibition, behavioral activation, and affective responses to impending reward and punishment: The BIS/BAS Scales. *J. Pers. Soc. Psychol.* **67**, 319–333 (1994).
4. Spielberger, C. D. *Manual for the State-Trait Anger Expression Inventory (STAXI).* Odessa, FL: Psychological Assessment Resources (1988).
5. Spielberger, C. D., Gorsuch, R. C., Lushene, R. E., Vagg, P. R. & Jacobs, G. A. *Manual for the State-Trait Anxiety Inventory*. Palo Alto, CA: Consulting Psychologists Press (1983).
6. Kahneman, D., Knetsch, J. L. & Thaler, R. Fairness as a constraint on profit seeking entitlements in the market. *Am. Econ. Rev.* **76**, 728–741 (1986).
7. Fischbacher, U. z-Tree: Zurich toolbox for ready-made economic experiments. *Experimental Economics* **10**, 171–178 (2007).
8. Holt, C. A. & Laury, S. K. Risk aversion and incentive effects. *Am. Econ. Rev.* **92**, 1644–1655 (2002).
9. Yamagishi, T. & Yamagishi, M. Trust and commitment in the United States and Japan. *Motivation and Emotion* **18**, 129–166 (1994).
10. Voracek, M., Manning, J. T. & Dressler, S.G. Repeatability and interobserver error of digit ratio (2D:4D) measurements made by experts. *Am. J. Hum. Biol.* **19**, 142–146 (2007).
11. Van Honk, J., Schutter, D. J., Bos, P. A., Kruijt, A.-W., Lentjes, E. G. & Baron-Cohen, S. Testosterone administration impairs cognitive empathy in women depending on second-to-fourth digit ratio. *Proc. Natl. Acad. Sci. USA* **108,** 3448–3452 (2011).
12. Eisenegger, C., Naef, M., Snozzi, R., Heinrichs, M. & Fehr, E. Prejudice and truth about the effect of testosterone on human bargaining behaviour. *Nature* **463**, 356–359 (2010).
13. Raudenbush, S.W. *Hierarchical Linear Models: Applications and Data Analysis Methods* (chapter 13) (Sage, 2002).
14. Kreps, D. M. & Wilson, R. Sequential equilibria. *Econometrica* **50**, 863–894 (1982).

15. Buskens, V. & Raub, W. Rational choice research on social dilemmas: Embeddedness effects on trust in *The Handbook of Rational Choice Social Research* (eds Wittek, R., Snijders, T. A. B. & Nee, V.) 113–150 (Stanford University Press, 2013).
16. Boksem, M. A. S. *et al*. Testosterone inhibits trust but promotes reciprocity. *Psychol. Sci.* **24**, 2306–2314 (2013).
17. Zethraeus, N., Kocoska-Maras, L., Ellingsen, T., Von Schoultz, B., Lindèn Hirschberg, A. & Johannesson, M. A randomized trial of the effect of oestrogen and testosterone on economic behavior. *Proc. Natl. Acad. Sci. USA* **106**, 6535–6538 (2009).
